# Unraveling CARD9 Mutations in Deep Dermatophytosis: A Genetic Gateway to Fungal Invasion and Immune Dysfunction

**DOI:** 10.3390/jof12060451

**Published:** 2026-06-21

**Authors:** Dipika Shaw, Gargi Mudey, Sunil Dogra, Hitaishi Mehta

**Affiliations:** 1 Department of Microbiology, Jawaharlal Nehru Medical College, Datta Meghe Institute of Higher Education and Research, Sawangi (Meghe), Wardha 442001, India; 2 Department of Dermatology, Venerology & Leprology, Postgraduate Institute of Medical Education and Research, Chandigarh 160012, India

**Keywords:** deep dermatophytosis, CARD9, dermatophytes

## Abstract

Deep dermatophytosis is a rare, life-threatening fungal infection characterised by the invasion of dermatophytes beyond the superficial layers of keratinised tissue into the dermis and subcutaneous tissues. The present review aimed to identify the current knowledge on the role of Caspase Recruitment Domain-containing protein 9 (CARD9) deficiency in the pathogenesis, clinical spectrum, diagnosis, and management of deep dermatophytosis. For innate immune activation, CARD9 acts as an adaptor molecule. Basically, CARD9 helps mediate the connection between the fungal pattern recognition receptor (Dectin-1) and the NF-κB and MAPK signalling pathways, and it mediates cytokine production, thereby activating phagocytic activities. Thereby, any change or mutation in the CARD9 gene may disrupt these pathways, leading to dysfunctional neutrophils and impaired Th17-mediated antifungal immunity. Clinically, patients with CARD9 deficiency are immunocompetent but susceptible to recurrent and/or severe fungal infections [*Candida*, dermatophytes (*Trichophyton* spp.), and phaeohyphomycetes]. Deep dermatophytosis in these patients is usually chronic, treatment-resistant, and characterized by erythematous papules, nodules, plaques, ulcers, or necrotic lesions, most of which occur on the lower limbs. It usually occurs in adulthood and is more common in males. There have been instances of geographic clustering of CARD9 deficiency in Asia, North Africa, and the Middle East. Early recognition and genetic diagnosis of CARD9 mutations in patients with recurrent or atypical deep dermatophytosis. Although antifungal therapy is essential, hematopoietic stem cell transplantation can be a definitive treatment for selected patients with CARD9 deficiency. Thus, CARD9 deficiency is a critical factor in the better management of patients but remains an underrecognized cause of severe, treatment-resistant deep dermatophytosis, and early genetic diagnosis is essential for guiding targeted management and improving patient outcomes. This review emphasises the importance of CARD9 in antifungal immunity and underscores the need for greater clinical awareness and the incorporation of genetic evaluation into the management of deep dermatophytosis.

## 1. Introduction

Dermatophytosis is the most common superficial fungal infection (SIF) caused by dermatophytes [1]. Dermatophytes are keratinophilic fungi confined to the keratinous tissue and invade keratinous material present in skin, nails and hair [2]. Generally, dermatophytosis (superficial) does not invade deeper layers, such as the dermis or subcutaneous tissue [3]. If left untreated or in immunocompromised individuals, the infection may extend beyond the epidermis, infiltrating deeper tissues and, in rare instances, spreading to internal organs, resulting in invasive dermatophytosis [3,4].

To date, several forms of invasive dermatophytic infections have been identified with unique clinical and pathological characteristics. Majocchi’s granuloma (MG) is characterized by the invasion of dermatophytes at perifollicular sites. It was first described by Domenico Majocchi in 1883, following a case involving a dermal granuloma caused by *Trichophyton tonsurans* [5]. Histopathological examination showed granulomatous inflammation centred around hair follicles. The condition was initially termed “granuloma tricofitico” and is now known as Majocchi’s granuloma [5]. Clinically, it manifests as nodular lesions affecting the hair follicles and the adjacent dermis [5,6]. Dermatophytic pseudomycetoma is a rare invasive fungal infection caused by dermatophytes. It typically affects the scalp, with fungal elements infiltrating the dermis or subcutaneous tissue.

It presents as tumour-like masses with fungal elements but lacks the sinus tracts and true granules characteristic of mycetoma [7]. Disseminated dermatophytosis is a rare and severe infection that occurs primarily in immunocompromised individuals and is characterised by fungal spread beyond the skin and subcutaneous tissue into internal organs, resulting in systemic disease [8]. Deep dermatophytosis is the invasion of fungi into the dermis, subcutaneous tissues, lymph nodes, and visceral organs beyond the epidermis [8]. The clinical manifestations of deep dermatophytosis include granulomatous lesions, hard nodules, lymphadenitis, and abscesses. The primary risk factors for deep dermatophytosis include underlying primary immunodeficiencies, and not exposure or secondary immunodeficiency [6]. CARD9 gene mutations have been recognised as a significant risk factor for susceptibility to deep and disseminated fungal infections, including candidiasis and dermatophytosis [9,10]. CARD9 is mainly expressed in myeloid cells and functions as a central adaptor molecule in the signal-ling pathways of C-type lectin receptors, which play a pivotal role in the recognition of fungal pathogens [11]. However, CARD9 deficiency is already recognized as a risk factor for severe infections caused by *Candida* spp. and *Aspergillus* spp. Infections and their association with deep dermatophytosis are also gaining recognition, especially in cases of severe and familial dermatophyte infections [12]. Thus, the present review focuses on CARD9 mutations, clinical spectrum, diagnostic approach, and therapeutic implications in deep dermatopytosis cases.

## 2. Clinical Spectrum and Etiological Agents of Deep Dermatophytosis

Deep dermatophytosis is more commonly reported in males, with a male-to-female ratio of 2.43:1. The average age of patients was 39.8 ± 16.6 years, and the most common age range was 8–83 years [13]. It is mainly observed in immunocompromised patients, accounting for 92.7% of all reported cases [6]. The lesions may take various forms, including papules, nodules, plaques, ulcers, pustules, abscesses, and tumours. The lesions are mainly found on the lower limbs (36.6%), followed by the trunk and scalp (17.1%), the genital area (14.6%), the face and neck (9.8%), and the upper limbs (4.9%) [6]. In immunocompromised individuals with deep dermatophytosis, the primary presentation was abscesses and cyst formation [14]. Documented in at least 46 cases by 2016, these lesions may be solitary or multiple and commonly involve the lower limbs, trunk, and upper limbs [15]. Their size varies widely, with cysts reaching up to 15 cm in diameter. Other than that, ulcerative lesions in deep dermatophytosis vary in size; while most are small (1–2 cm) and covered with thick crusts, some can enlarge significantly, forming giant ulcers up to 15 cm in diameter [16]. In rare cases, tinea corporis-like circinate and pustular plaques have also been observed, especially in HIV-positive individuals [17]. Rapid dissemination with numerous nodular lesions may occur in severely immunocompromised hosts [18].

Lesions in cases of deep dermatophytosis often present as indurated nodules, plaques, or abscess-like swellings that can mimic cutaneous tuberculosis, deep fungal infections (such as sporotrichosis and chromoblastomycosis), and atypical mycobacterial infections. The infiltrated violaceous plaques in cases of cutaneous lymphoma or Kaposi sarcoma may raise concern, especially in HIV-positive individuals [19,20]. Pyoderma gangrenosum and sarcoidosis may also be included in the differential diagnosis when ulceration or granulomatous features predominate. The other presentations, such as umbilicated nodules, can mimic cryptococcosis and molluscum contagiosum, while papules and nodules may resemble acute perforating collagenosis [21,22]. Lesions often appear as cellulitis-like plaques, similar to Majocchi’s granuloma (MG), leading to misdiagnosis and unnecessary antibiotic use [6]. Thus, the high level of suspicion and approach, combined with appropriate histopathology and fungal stains, is crucial for an accurate diagnosis. Therefore, the significant clinical differentials of deep dermatophytosis, highlighting distinguishing features that aid in diagnosis, are summarised in Table 1.

The etiological agents of deep dermatophytosis include dermatophyte species such as *Trichophyton rubrum*, *T. mentagrophytes*, *T. violaceum*, *T. schoenleinii*, *Microsporum audouinii*, and *M. ferrugineum* [9,10,12,23,24,25,26,27,28,29,30].

## 3. Immunological Mechanism and Biological Immune Regulatory Role of CARD9

CARD9 is a key cytosolic adaptor protein essential for antifungal immunity, linking C-type lectin receptors (PRRs) to downstream signalling pathways that activate innate and adaptive immune responses against fungal pathogens [31]. CARD9 is a key player in both innate and adaptive immune responses, especially against fungal, bacterial, and viral infections [32]. It is expressed in myeloid cells (dendritic cells and macrophages) and non-myeloid cells, such as cardiomyocytes and endothelial cells [33]. CARD9 has a broad expression profile in various organs, including the spleen, lungs, heart, brain, peripheral blood, and bone marrow [33,34,35].

The immune system first identifies fungal cell wall components (such as β-glucan) via C-type lectin receptors (CLRs) [Dectin-1, Dectin-2, Dectin-3, Mincle, and Mannose Receptor (CD206)]. Among these, Dectin-1 is the most extensively studied receptor, activating the CARD9-mediated intracellular signalling pathway upon binding to β-glucan, the predominant component of the fungal cell wall. Upon recognition of the fungal component by the PRRs, the Syk kinase is activated, leading to the assembly of the CBM signalosome (CARD9-BCL10-MALT1) [36]. This process depends on Vav proteins (Vav1, Vav2, and Vav3), which are essential for effective downstream signalling [31]. Deletion of Vav proteins in mice causes severe antifungal immune defects, similar to those observed in CARD9-deficient mice, highlighting their critical role in CARD9-mediated immune signalling pathways [37].

**Figure 1:**
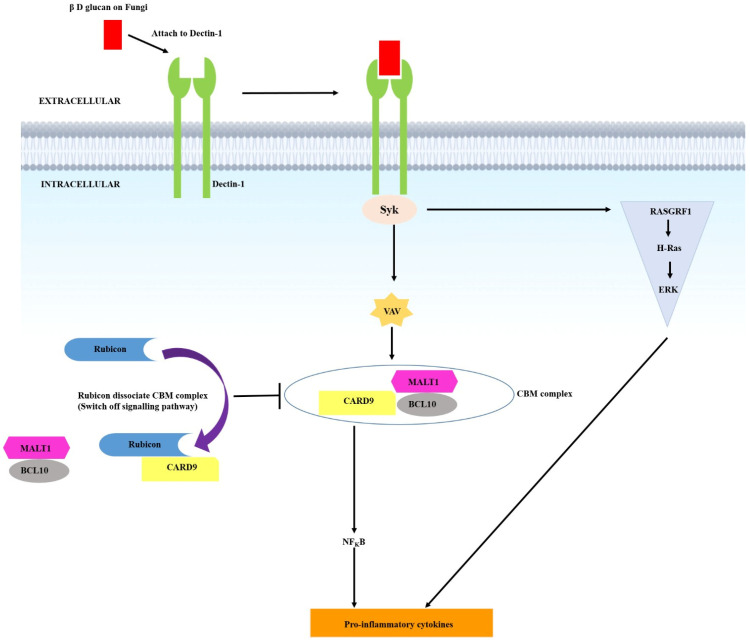
Schematic representation of CARD9 signalling pathway for production of pro-inflammatory cytokines. Upon ligand binding, Dectin-1 activates Syk, leading to the assembly of the CARD9-BCL10-MALT1 (CBM) complex. VAV enhances CARD9 activity. Rubicon negatively regulates the pathway by disrupting the CBM complex.

CARD9 activation initiates signalling cascades, such as NF-κB which increase the inflammatory response. The ERK pathway is activated through a RASGRF1–H-Ras-dependent mechanism, while NF-κB activation occurs via the CBM (CARD9–BCL10–MALT1) complex [38]. Therefore, this signalling pathway ultimately leads to the release of various pro-inflammatory cytokines (IL-6, IL-12, GM-CSF, TNF, IL-1β and GM-CSF) as well as chemokines including CXCL1, CXCL2, and CXCL8, which are essential for recruiting and activating immune cells, thereby supporting the antifungal defence mechanism and shaping the adaptive immune response (Figure 1) [31,33]. Rubicon acts as a negative regulator of CARD9 signalling by inhibiting the CBM complex, thereby inhibiting NF-κB activation and downstream pro-inflammatory cytokine responses [31].

The other functions of CARD9 include regulating differentiation and polarisation into M1/M2 macrophages and have been implicated in tumour metastasis [33]. CARD9 also regulates neutrophil recruitment to sites of infection and enhances antigen presentation and T cell responses in DCs [33]. In innate lymphoid cells, it supports the proliferation of intestinal epithelial cells [33]. In non-immune cells, CARD9 regulates cardiomyocyte survival by promoting autophagy and inhibiting apoptosis, and regulates responses to shear stress in endothelial cells. Further, in microglia, CARD9 also simulates the secretion of inflammatory cytokines [33]. Therefore, CARD9 leads to the release of cytokines by activating macrophages, thereby causing oxidative stress and tissue dysfunction. Thus, it exacerbates inflammation and injury, suggesting a role in immune regulation and the pathogenesis of inflammatory disease.

In dermatophyte infections, CARD9 plays a crucial role in synchronizing antifungal immunity by regulating neutrophil recruitment, macrophage activation, and dendritic cell–mediated T-cell responses, thereby driving Th17-mediated immunity and cytokine release [31,36,39,40]. Therefore, Th17 cells and associated cytokines are critical for protection against both superficial and invasive fungal infections [40,41,42].

Mutations in the CARD9 gene disrupt these signaling pathways, leading to impaired cytokine production, defective neutrophil recruitment, and compromised antifungal immunity [31,43]. This immune dysfunction predisposes affected individuals to chronic, recurrent, and invasive dermatophyte infections, including deep dermatophytosis [3,9].

CARD9 Deficiency- It is critical for pulmonary immunity, as its deficiency results in reduced accumulation of IFN-γ-producing memory T cells and increased susceptibility to *Cryptococcus neoformans* [44]. It is also critical in fighting dematiaceous fungi, as its deficiency leads to inefficient immune cell infiltration and lower cytokine production, highlighting its general role in antifungal immunity [45]. It acts downstream of C-type lectin receptors, together with BCL10 and MALT1, in the activation of inflammatory cytokine production. In respiratory fungal infections, it acts sequentially with MyD88. MyD88 mediates initial chemokine responses, while CARD9 maintains inflammation and efficient fungal clearance [46]. CARD9 is therefore a critical component of human immunity, as mutations in CARD9 make an individual more prone to severe fungal infections by impairing the connection between pathogen recognition, immune activation, and the maintenance of fungal control.

The importance of CARD9 is cell type-dependent, especially in myeloid cells (neutrophils, macrophages, and dendritic cells), which exhibit varying levels of CARD9 dependence for NF-κB activation [38,47]. Neutrophils play a significant role in protecting against systemic *Candida albicans* infection [31,48]. Although most neutrophil functions, including phagocytosis, ROS production, and chemotaxis, are CARD9-independent [31], the killing of unopsonized yeast requires CARD9 in a PI3Kγ-dependent manner [49]. This cell-type-specific deficiency helps explain the increased susceptibility to fungal infections in the CNS of CARD9-deficient patients, in whom opsonization is minimal. It is important to note that neutrophils can still kill *Candida*. CARD9 also promotes neutrophil migration into infected tissues by inducing CXC chemokines, which helps to explain the organ-specific fungal susceptibility observed in patients with CARD9 mutations [39,50].

The CARD9 signalling pathway also supports Th17 cell differentiation by inducing the secretion of IL-1β, IL-6, and IL-23 [31]. Th17 cells, in turn, secrete IL-17 and IL-22, which play crucial roles in antifungal immunity by recruiting neutrophils and activating epithelial cells to secrete antimicrobial peptides. CARD9 deficiency impairs both Th1 and Th17 immunity, leading to decreased circulating Th17 cell numbers and increased susceptibility to mucocutaneous fungal infections [31]. The Dectin-1/Syk/CARD9 signalling pathway is more potent than TLR signalling pathways in triggering Th17 immunity, which may be attributed to the production of higher amounts of IL-23 than IL-12 by Dectin-1-stimulated dendritic cells [40]. CARD9-mediated Th17 immunity is protective and contributes to the pathogenesis of autoimmune and allergic diseases [11]. Additionally, Dectin-1 and TLR2 can work together to promote IL-12 release, which will aid in the formation of Th1 cells. IFN-γ is secreted by Th1 cells, activating macrophages and neutrophils to eradicate systemic fungal infections [33].

***Genetic Insights into CARD9 Mutations on Deep Dermatophytosis*-** In case of invasive fungal infections, Bi-allelic loss-of-function mutations noted in the CARD9 genes [9]. CARD9 is crucial for antifungal immunity, particularly by mediating signalling pathways in myeloid cells, such as macrophages and dendritic cells. When this adaptor protein is defective, the immune system fails to respond to specific fungi [11]. As a result, affected individuals are particularly susceptible to infections caused by *Candida* species, dematiaceous moulds, and, in some cases, dermatophytes.

**Figure 2:**
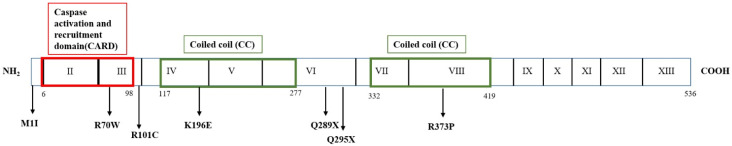
Schematic representation of CARD9 mutation in deep dermatophytosis.

CARD9 is a unique intracellular adaptor protein and a member of the CARD (Caspase-associated Recruitment Domain) family, characterised by the presence of a CARD domain involved in mediating protein–protein interactions during immune signalling [33]. The CARD9 gene is located on chromosome 9q34.3 and comprises 13 exons [43]. The approximate molecular size of CARD9 was 62.3 kDa, and its full-length cDNA consists of 2108 base pairs and encodes a protein of 536 amino acids [51]. Structurally, CARD9 shares similarities with CARMA (CARD-containing MAGUK) family proteins but notably lacks the C-terminal MAGUK domain [52]. Instead, CARD9 consists of two central functional regions: an N-terminal CARD domain (amino acids 7–98) and a C-terminal coiled-coil domain (amino acids 140–420) [53,54] (Figure 2).

***Geographic Distribution and Consanguinity***- CARD9 deficiency-related deep dermatophytosis and invasive fungal infections have been documented worldwide, with a preponderance of cases in North Africa and the Middle East [9]. The most significant number of cases was reported in Algeria [9,23], Tunisia [9], Morocco [9], and Iran [10], suggesting a geographical hotspot for these cases. Cases of deep dermatophytosis were documented in Turkey [12,55], China [26,27,30], Brazil [24], Egypt [28], Japan [25], and the United States of America [50], indicating its global, although unrecognised, spread. Notably, a high incidence of consanguineous marriages has been observed in most of these cases, particularly in patients from Algeria, Iran, Tunisia, and Morocco, where consanguineous marriages are a cultural norm [9]. Of the 31 cases, 26 had a proven history of consanguineous marriage. Hence, this provides definitive evidence for the autosomal recessive pattern of inheritance of CARD9 gene mutations. It helps in genetic counselling and screening in consanguineous families, particularly in endemic areas (Table 2 and Supplementary Table S1).

*Clinical Spectrum and Pathogens-* The clinical presentation of CARD9 deficiency is wide-ranging, and patients usually present in early childhood or adolescence. Yet, some patients, particularly those with heterozygous mutations or mild forms, may have an onset of symptoms in adulthood. The infections are usually chronic, recurrent, and multifocal, involving the skin, nails, scalp, mouth, lymph nodes, and, in some instances, the central nervous system (CNS) or bone [9]. The most commonly isolated causative fungal pathogens are dermatophytes, particularly *Trichophyton rubrum*, *T. violaceum*, *T. mentagrophytes*, *T. tonsurans*, *T. verrucosum*, and *Microsporum ferrugineum* [9,10,12,23,24,25,26,27,28,29,30]. It is important to note that, in most instances, patients are colonised by multiple dermatophyte species simultaneously, indicating the host’s inability to clear the pathogens and thereby failing to elicit an efficient immune response (Table 2 and Supplementary Table S1).

In addition to dermatophytes, *Candida albicans* and other *Candida* spp. were involved in mucocutaneous candidiasis (primarily oral and vaginal thrush). At the same time, *Aspergillus fumigatus*, *Aspergillus flavus*, and *Mucor irregularis* were identified in some patients with systemic involvement or invasive fungal disease (e.g., mucormycosis and aspergillosis). Rare coinfections with *Malassezia furfur* were also observed [9,12,27,29,50].

***Genotype-Phenotype Correlation-*** Mutations of the CARD9 gene result in an intrinsic functional defect in the innate antifungal immune response, especially impeding Th17-mediated pathways. The mutations reported in these patients are missense and nonsense mutations and generally lead to loss of function. It has been considered as a hotspot mutation, common among North African and Middle Eastern patients, which is c.C865T (pQ289X) [c indicates coding DNA (cDNA) sequence and p indicate protein (amino acid) change] [9,23]; that lies in exon 6. This mutation is highly linked to severe dermatophytosis, predominantly affecting skin, nails and lymph nodes, and with CNS disease in the most severe cases. Other mutations include c.C301T (R101C) [9], c.302G>T (R101C) [24], c.596A>R (K196E) [25,26], c.208C>T (R70W) [12], c.3G>C (M1I) [50], c.883C>T (Q295X) [10], and compound heterozygous or intronic mutations like c.1269+18G>A and c.184+5G>T [29]. Notably, heterozygous mutations (e.g., Y91H, Q295X) [29] were generally associated with later or milder onset, whereas homozygous mutations predominated in severe, disseminated infections. There seems to be a genotype-phenotype association: Q289X is associated with dermatophytosis and lymph node involvement, whereas R101C is associated with osseous and systemic dissemination. Patients with compound mutations frequently had coinfections (e.g., mucormycosis and candidiasis) and poor treatment responses (Table 2 and Supplementary Table S1).

***Treatment and Outcome-*** The treatment of fungal infections in CARD9-knockout patients is complicated and usually ineffective with conventional antifungal drugs alone. The antifungal drugs used included griseofulvin, fluconazole, itraconazole, terbinafine, and newer drugs such as voriconazole, posaconazole, and liposomal amphotericin B. In some cases, the drugs were used alternately or in combination based on treatment failure. Specifically, patients with Q289X mutations needed itraconazole, with variable success. Patients with resistant or widespread fungal infections needed combination therapy (e.g., terbinafine + voriconazole + IFN-γ). Some patients received systemic corticosteroids or interferon-γ as adjuncts to enhance the immune response, with some success. The outcome was variable: Surviving patients were mostly those who received early diagnosis and effective antifungal treatment. Death occurred in patients with CNS involvement, late diagnosis, or ineffective treatment, particularly in patients treated before the discovery of CARD9 deficiency [6,9].

## 4. Diagnostic Tools for Deep Dermatophytosis

**Figure 3:**
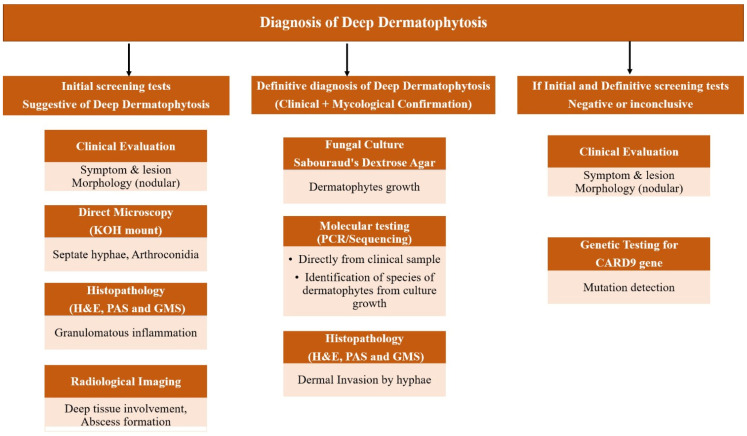
Diagnostic Algorithm for deep dermatophytosis cases. KOH—Potassium Hydroxide, H&E—Hematoxylin and Eosin, PAS—Periodic Acid–Schiff, GMS—Grocott’s Methenamine Silver, PCR—Polymerase chain reaction, CARD9—Caspase Recruitment Domain-containing protein 9.

Deep dermatophytosis involves the deeper tissues of the skin (dermis and subcutis), lymph nodes, and, occasionally, deeper organs. It is pertinent to note that it has been reported in immunocompromised patients, particularly those with CARD9 mutations, solid organ transplants, hematologic malignancies, or HIV/AIDS. Because of its unusual clinical presentation and the involvement of deeper tissues, the diagnosis of deep dermatophytosis involves a differential diagnosis and an amalgamation of clinical, histopathological, microbiological, and molecular approaches (Table 3 and Figure 3).

***Clinical Assessment***- Deep dermatophytosis usually presents as subcutaneous nodules, indurated plaques, abscesses, or lymphadenopathy [6,56]. The lesions are chronic and resistant to topical treatment. Immunocompromised patients, especially those with CARD9 mutations, are more prone to infection, which can be either localized or disseminated. The clinical presentation varies according to the immune status and site of involvement and may include papules, nodules, plaques, ulcers, pustules, abscesses, or tumoral lesions [6] (Table 4). Due to its rarity and nonspecific features, high clinical suspicion is crucial in endemic regions or patients with recurrent or atypical dermatophytic presentations.

**Histopathological and Cytological examination**- For identification of fungal elements in the dermal layer, various specialised stains such as Periodic Acid-Schiff (PAS), Gomori Methenamine Silver (GMS), Haematoxylin and Eosin (H&E), May-Grünwald-Giemsa, and Papanicolaou can be used [59,60,61,62]. Parakeratosis, basket-weave keratin, spongiosis, neutrophilic infiltration in the basal layers of the epidermis, eosinophils in the dermis, acanthosis, and hyperkeratosis are essential histopathological features for the diagnosis of deep dermatophytosis [61,62]. However, these special stains require technical skill and may not be readily available in clinical practice [60]. Another method is cytological examination by imprint smear or fine-needle aspiration, which may reveal grain production or the Splendore-Hoeppli reaction, suggestive of deep fungal infection [59].

**Direct Microscopy**- Direct microscopy with potassium hydroxide (KOH) mounts, with or without calcofluor white staining, is a simple, rapid, and inexpensive technique for detecting fungal components, such as hyphae or arthroconidia, in skin scrapings or biopsies [63]. KOH is effective for detecting superficial dermatophytosis. Still, its role in detecting deep dermatophytosis is significantly reduced because the dermatophytes reside in deeper skin layers in this condition. The fungal tissue can be detected under fluorescence microscopy, but may still miss deeper fungal elements [6]. Despite these limitations, the KOH mount remains a useful preliminary diagnostic tool when combined with histopathological examination and fungal culture.

**Fungal culture-** Culturing biopsy tissue or aspirated material is vital for identifying the causative agent in deep dermatophytic infection. Sabouraud Dextrose Agar (SDA), supplemented with antibiotics such as chloramphenicol (0.05%) and cycloheximide (0.5%), is routinely used to promote dermatophyte growth while suppressing bacterial and saprophytic fungal growth [63]. Dermatophyte growth may take 1 to 4 weeks, and identification relies on evaluating macroscopic and microscopic features. Although growing organisms by the conventional method is time-consuming, it remains an essential method for definitive species identification and for performing antifungal susceptibility testing to guide appropriate, targeted therapy [6].

**Molecular Method-** In situations of uncertain diagnosis, especially in immunosuppressed patients, it is essential to identify the fungal species correctly. Conventional PCR and real-time PCR are fast and reliable approaches for species-level identification [63]. qPCR is particularly helpful for directly detecting fungal DNA in tissue samples from culture-negative deep dermatophytosis, thereby significantly improving diagnostic sensitivity [6,64]. Thus, molecular identification directly from clinical specimens (tissue samples) enables rapid detection of the responsible aetiology, rather than the time-consuming conventional culture method. Therefore, provide timely, species-specific identification to initiate appropriate antifungal therapy, which is essential for the effective management of invasive dermatophyte infections.

**Imaging-** Radiological imaging, although non-specific, may help assess the extent of deep dermatophytosis, especially when there is suspicion of bone or soft-tissue involvement. Although primarily used for diagnosing eumycetoma, ultrasound and MRI help evaluate the extent of soft-tissue involvement in invasive dermatophyte infections. MRI is considered more sensitive and specific than CT in detecting early changes and assessing treatment response. The characteristic “dot-in-circle” sign, as seen in mycetoma, may also help occasionally in differential diagnosis. CT scan imaging can be used when there is a suspicion of systemic dissemination. Radiological imaging supports clinical and histopathological diagnoses of deep dermatophytosis [6].

**Immunological test-** Flow cytometry-based intracellular cytokine staining and ELISA are valuable diagnostic tools for detecting Th17-related immune defects in deep dermatophytosis. Stimulated CARD9-deficient patients display decreased percentages of IL-17A+CD4+ (Th17) and IL-22+IL-17-CD4+ (Th22) cells. It is associated with reduced levels of IL-17A, IL-22, and IFN-γ, suggesting a defect in Th17 and Th22 responses. It is due to a defect in the production of pro-inflammatory cytokines such as IL-6, IL-1β, and TNF-α. The increased levels of serum IgE and eosinophils, in the presence of a STAT3 mutation, indicate a bias towards Th2-mediated immune responses. These observations argue for the importance of immunophenotyping and cytokine analysis as diagnostic tools for identifying defects in the Th17 pathway in invasive fungal infections [9].

**Genetic Testing for CARD9 Mutations-** Genetic testing for CARD9 mutations is essential in patients with deep dermatophytosis. Located on chromosome 9, the CARD9 gene contains 13 exons and encodes a signalling protein vital for antifungal immune defence. Mutational analysis of CARD9 can be performed by Sanger sequencing or whole-genome sequencing to detect mutations in its exons and introns. Thus, it will help in confirming the mutations responsible for CARD9 deficiency [9,24,25].

## 5. Implications for Diagnosing Underlying Immunodeficiency

A high level of suspicion is required for the identification of deep or invasive dermatophytosis based on the clinical presentation, and also needs to determine the close link between invasive infections and the host’s immune system, especially CARD9 pathway mutations. Patients with extensive, chronic, and treatment-refractory dermatophytosis should be suspected of having CARD9 deficiency, especially when the disease involves multiple body sites, including the skin, nails, and lymph nodes. There are various warning signs for the detection of invasive or deep dermatophytosis, including family history of similar fungal infections, early childhood presentation, consanguineous parents, and failure to respond to systemic antifungal therapy. Frequent or simultaneous cases of deep fungal infections, such as candidiasis or mucormycosis, may also point towards primary immunodeficiency. In the absence of known immunosuppressive conditions, the involvement of deep tissues, including the lymph nodes, central nervous system, or bones, should also suggest the possibility of CARD9 mutations.

## 6. Treatment Strategy for CARD9 Deficiency in Deep and Disseminated Dermatophytosis

Deep and disseminated dermatophytosis requires a personalized, aggressive treatment strategy that may include systemic antifungal therapy, immune status evaluation, and, in some cases, surgery. The treatment of this condition is not evidence-based but instead relies on clinical expertise and new literature, especially in immunocompromised patients. Table 5 lists the current treatment modalities for severe and unresponsive disease.

## 7. Research Gaps and Future Directions

To identify the exact prevalence of deep dermatophytosis cases, a cost-effective genetic screening strategy is needed among endemic regions such as Algeria, Tunisia, Iran, Brazil, Turkey, the USA, and Egypt. Initially, screening priority must be given to high-risk groups such as consanguineous families and patients with deep fungal infections. However, large-scale pheno-genotypic studies are necessary to understand the role of CARD9 mutations in disease severity. Genetic analysis using next-generation sequencing and Sanger sequencing can help detect additional immunodeficiencies associated with fungal infections. In addition to CARD9 analysis, evaluating cytokine profiles, histopathological findings, and fungal cultures can aid in diagnosing deep dermatophytosis. These diagnostic approaches may support the development of standardised treatment protocols that integrate antifungal agents, immunotherapies, and emerging immunomodulators or gene therapies. Consequently, collaborative clinical trials and research efforts are essential to identify previously undiagnosed cases and to guide the formulation of effective therapeutic strategies.

## 8. Conclusions

Deep and disseminated dermatophytosis usually develops in the immunocompromised host. Therefore, genetic susceptibility to CARD9 deficiency should be systematically assessed. An accurate diagnosis can only be made with a combination of clinical suspicion, rigorous mycologic work-up, and advanced immunochemical testing. While culture and histopathology remain the cornerstones of diagnosis, molecular diagnostics and CARD9 gene analysis are gaining importance. Defects in host immunity play a crucial role in the pathogenesis of fungal diseases. CARD9 mutations highlight the importance of understanding genetic susceptibility to fungi. Early diagnosis, the application of advanced diagnostic techniques, and tailored treatment are key to adequate disease control of this frequently neglected entity to lower its severity.

## Figures and Tables

**Table 1:** Differential Diagnosis of Deep Dermatophytosis.

Condition	Key Clinical Features	Distinguishing Clues
Cutaneous Tuberculosis (Lupus vulgaris)	Chronic plaques or nodules, often reddish-brown, may ulcerate	Positive Mantoux test, caseating granulomas on histopathology, AFB staining positive
Cutaneous Leishmaniasis	Papules or ulcers, endemic regions, may crust or ulcerate	Leishmania donovani bodies in smear/biopsy, positive rK39 serology
Sporotrichosis	Subcutaneous nodules, often ulcerative, may follow lymphatic channels (nodular lymphangitis)	Cigar-shaped or oval yeast forms on histology; positive culture on Sabouraud agar; may show lymphatic spread; PCR available
Atypical Mycobacterial Infection	Chronic nodules, plaques, or abscesses; may show sporotrichoid pattern; common on extremities or trauma sites	Acid-fast bacilli on Ziehl–Neelsen stain, PCR for atypical mycobacteria
Chromoblastomycosis	Chronic verrucous plaques or nodules, often on lower limbs; may ulcerate or become cauliflower-like	Sclerotic (Medlar) bodies on histology (brown, copper-penny–like), slow progression, endemic in tropics
Eumycetoma	Tumefaction with draining sinuses and grains; typically affects feet or lower legs (“Madura foot”)	Presence of fungal grains (black/white), dot-in-circle sign on MRI/ultrasound, progressive bone involvement
Kaposi Sarcoma	Violaceous nodules/plaques, typically in immunosuppressed (HIV) patients	Spindle cells on biopsy, HHV-8 immunohistochemistry positive
Pyoderma gangrenosum	Painful ulcers with undermined edges; associated with IBD or autoimmune conditions	Diagnosis of exclusion, histopathology shows neutrophilic infiltration without infectious agents
Sarcoidosis	Papules or plaques; may mimic granulomatous infection	Non-caseating granulomas on biopsy, systemic signs (e.g., lung involvement, ACE levels)

ACE—Angiotensin-Converting Enzyme, AFB—Acid-Fast Bacilli, HIV—Human Immunodeficiency Virus, HHV-8—Human Herpesvirus 8, IBD—Inflammatory Bowel Disease, MRI—Magnetic Resonance Imaging, PCR—Polymerase Chain Reaction, rK39—Recombinant K39 Antigen (used in serodiagnosis of visceral leishmaniasis).

**Table 2:** Summary of Deep dermatophytosis cases and CARD9 gene mutation status [9,10,12,23,24,25,26,27,28,29,30,50,55].

Parameter	Key Findings
Time period	2009–2024
Geographical distribution	North Africa (Algeria, Morocco, Tunisia), Middle East (Iran, Turkey), Asia (China, Japan), sporadic cases in USA, Brazil, Egypt
Common clinical presentations	Tinea corporis (most frequent), often with onychomycosis, tinea capitis and tinea cruris
Mutation type	Predominantly homozygous mutations; few heterozygous cases
Consanguinity	Frequently reported, especially in North Africa and the Middle East
Most common mutations (with nucleotide changes)	Q289X (c.865C>T/c.883 C>T)—most frequent, R101C (c.301C>T/c.302G>T), K196E (c.586A>G/c.596A>R)
Other reported mutations (with nucleotide changes)	R70W (c.208C>T); M1I (c.3G>C); Q295X (c.883C>T); R317R/c. 184+5G>T; c.271T>C Y91H, c.1269+18G>A;
Mutation nature	Mostly nonsense and missense mutations causing loss of function
Exon involvement	Commonly- exons 3, 4, and 6 Less common- 8 and intronic 8
Clinical outcome	Majority alive with chronic/recurrent infection; some fatal cases reported
Genetic pattern	Autosomal recessive inheritance

c—Coding DNA sequence.

**Table 3:** Diagnostic Tools for Deep Dermatophytosis.

Tool	Sample Type	Target/Detection	Advantages	Limitations
Clinical evaluation	Clinical presentation	Symptoms and lesion morphology	First clue to diagnosis	Non-specific; mimics other diseases
Direct Microscopy (KOH, CFW)	Skin/tissue scrapings	Hyphae/arthroconidia	Rapid, inexpensive	Low sensitivity in deep infections
Histopathology (H&E, PAS, GMS)	Skin/tissue biopsy	Tissue invasion by fungal elements	Demonstrates invasion	Cannot identify species
Culture on SDA	Biopsy, aspirate	Viable dermatophyte growth	Gold standard for species isolation	Time-consuming; may be negative in deep tissue
PCR and Sequencing	Biopsy/tissue DNA	Dermatophyte DNA (ITS regions)	Fast, specific, detects non-culturable fungi	Requires expertise and facilities
Radiological Imaging	Deep tissue/organs	Structural involvement	Evaluates disease extent	Non-specific; cannot confirm fungal etiology
Genetic testing (CARD9 mutation)	Blood (genetic material)	CARD9 gene mutation	Identifies underlying predisposition	Not diagnostic; only for select cases

KOH—Potassium Hydroxide, CFW—Calcofluor White, H&E—Hematoxylin and Eosin, PAS—Periodic Acid–Schiff, GMS—Grocott’s Methenamine Silver, SDA—Sabouraud Dextrose Agar, PCR—Polymerase chain reaction, ITS—Internal Transcribed Spacer, CARD9—Caspase Recruitment Domain-containing protein 9.

**Table 4:** Clinical Presentation differentiating with other invasive dermatophytosis [6,57,58].

Feature	Majocchi’s Granuloma	Deep Dermatophytosis	Pseudomycetoma	Disseminated Dermatophytosis
**Definition**	Dermatophyte infection extending into hair follicles and perifollicular dermis	Dermatophyte invasion into deep dermis/subcutis, may involve regional lymph nodes	Tumor-like subcutaneous lesion caused by dermatophytes from hair shafts; resembles eumycetoma	Systemic spread of dermatophytes to multiple skin sites or internal organs (e.g., CNS, lymph nodes, bone)
**Immune Status**	Typically immunocompetent or local immunosuppression (e.g., topical steroids)	Often linked to immunodeficiency (CARD9, HIV, SLE); sometimes in immunocompetent	Seen in immunocompromised or genetically predisposed individuals	Occurs in severe immunosuppression (advanced HIV, transplants, CARD9 deficiency)
**Pathogens**	*Trichophyton rubrum*, *T. mentagrophytes*	*Trichophyton* spp. (esp. *T. rubrum*, *T. mentagrophytes*)	*T. violaceum*, *T. rubrum*	*T. rubrum*, occasionally *Microsporum gypseum*
**Clinical Presentation**	Follicular papules, pustules, or nodules on limbs, thighs, or beard area	Nodules, plaques, or granulomas; regional lymphadenopathy possible	Firm nodules, typically on scalp or limbs; no sinus tracts	Multisite nodules, systemic symptoms (fever, malaise), CNS/bone/visceral involvement
**Histopathology**	Perifollicular granulomatous inflammation with hyphae in hair shafts	Granulomatous inflammation with hyphae in deep dermis or subcutis	Granulomatous reaction with fungal hyphae in granule-like aggregates (pseudo-grains)	High fungal burden; fungal elements in organs; angioinvasion, necrosis
**Mortality Risk**	Low	Moderate (~28% in lymph node or organ-involved cases)	Low to moderate	High—due to systemic complications (e.g., sepsis, CNS involvement)
**Treatment**	Oral antifungals (terbinafine, itraconazole); shorter duration typically sufficient	Prolonged systemic antifungals; surgical excision in localized cases	Long-term antifungal therapy ± surgical excision	Aggressive systemic antifungals + treatment of immunosuppression; high relapse risk

CARD9—Caspase Recruitment Domain-containing protein 9, HIV—Human Immunodeficiency Virus, SLE—Systemic Lupus Erythematosus, CNS—Central nervous center.

**Table 5:** Treatment Strategies for Deep and Disseminated Dermatophytosis [41,42,65].

Category	Agent/Approach	Dosage/Use	Notes
**First line- Systemic Antifungals**	**Itraconazole**	200–400 mg/day (divided doses)	Broad-spectrum; lipophilic; good dermal penetration
	**Terbinafine**	500 mg/day	Fungicidal; preferred for recalcitrant cases; emerging resistance in some regions
	**Fluconazole**	150–300 mg/week	Less effective; option if first-line agents are contraindicated
	**Griseofulvin**	250–500 mg twice daily (with fatty meals)	Older agent; limited efficacy; still used in some settings
	**Amphotericin B**	IV dosing (based on formulation)	Reserved for life-threatening systemic or CNS involvement
**Second line- Combination Therapy**	**Dual systemic**	e.g., Terbinafine + Itraconazole	Consider in resistant cases
	**Systemic + topical**	Oral antifungal + topical azole/allylamine	Enhances skin clearance, reduces superficial reservoirs
**Adjunctive Measures**	**Surgical excision**	Case-dependent	For localized nodules, pseudomycetoma, or abscesses
	**Liver function tests**	Baseline + monthly	Essential with itraconazole and prolonged terbinafine use
**Immunomodulation**	**Interferon-gamma (IFN-γ)**	Subcutaneous (off-label)	Enhances Th1 responses; helpful in CARD9 deficiency
	**GM-CSF**	Dosing varies	Boosts neutrophil/monocyte function; used in selected refractory cases
	**Adoptive T-cell or NK cell therapy**	Experimental	Promising in invasive fungal infections; not yet established in dermatophytosis
	**TLR agonists/AMPs**	Preclinical/experimental	Potential for future adjuncts in enhancing innate antifungal defense
**Prognosis & Precautions**	**Avoid steroids**	Topical/systemic	Risk of dissemination and delayed diagnosis
	**Survival rate**	100% in cutaneous-only cases; ~28% mortality in systemic disease	Prompt antifungal therapy crucial
	**Pediatric/pregnancy considerations**	Prefer topicals; fluconazole safer than itraconazole	Systemic agents used cautiously; specialist input advised

AMPs—Antimicrobial Peptides, CNS—Central Nervous System, GM-CSF—Granulocyte-Macrophage Colony-Stimulating Factor, IFN-γ—Interferon-gamma, IV—Intravenous, LFTs—Liver Function Tests, NK—Natural Killer, TLR—Toll-like Receptor.

## Data Availability

No new data were created or analyzed in this study. Data sharing is not applicable to this article.

## Supplementary Materials

The following supporting information can be downloaded at: https://www.mdpi.com/article/10.3390/jof12060451/s1, Supplementary Table S1. Deep dermatophytosis cases and CARD9 gene mutation status.
