# Supplementary material for: Unraveling CARD9 Mutations in Deep Dermatophytosis: A Genetic Gateway to Fungal Invasion and Immune Dysfunction
Source: J Fungi (Basel). 2026 Jun 21;12(6):451. doi: 10.3390/jof12060451 (PMC13301113; doi:10.3390/jof12060451)
Supplement: Supplementary file 1 [file jof-12-00451-s001.zip › jof-4188081-supplementary.pdf]

**Supplementary Table S1. Deep dermatophytosis cases and CARD9 gene mutation status.**

| Sl no | Year of article published | Pedigree* | Country | Consanguineous marriage | Age of Onset | Year of Last Follow up | Sex | Infection site as per dermatophytic infection | Dermatophytic clinical diagnosis | Dermatophyte etiology | Other infection site | Other than tinea diagnosis | Pathogen 1 | Co-morbidity | Homozygous/Heterogygous mutation | Nucleotide change | CARD9 gene mutation (p) | CARD9 gene muattion exon no | Treatment | Status | References |
|---|---|---|---|---|---|---|---|---|---|---|---|---|---|---|---|---|---|---|---|---|---|
| 1 | 2024 | NA | Japanese | NA | 80 | NA | M | Skin | Tinea corporis | TR | NA | NA | NA | NA | Homozygous | c.586 A > G | K196E | 4 | NA | NA | [25] |
| 2 | 2022 | NA | China | NA | 37 | 38 | M | Skin | Tinea corporis | TT | Tinea versicolor | NA | NA | NA | Homozygous | c.596A>R | K196E | 4 | Terbinafine, itraconazole, voriconazole and posaconazole | NA | [26] |
| 3 | 2020 | NA | China | No | 17 | 48 | M | Skin | Tinea corporis | TR | Oral, nasal | Candidiasis, Mucormycosis | CA, MI | NA | Heterozygous | R317R/c. 184 + 5G> T | | NA | Itraconazole and terbinafine | NA | [27] |
| 4 | 2020 | NA | USA | NA | 16 | 31 | M | Skin, nail | Tinea corporis, Onychomycosis | TR, TV | NA | NA | AF, Afl | NA | Heterozygous | c.271T>C Y91H, c.1269+18G>A | | 3, intronic 8 | initially griseofulvin and subsequently ketoconazole then itraconazole and posaconazole and then amphotericine B | Alive | [29] |
| 5 | 2019 | NA | China | No | 16 | 27 | F | Skin | Tinea corporis | MF | NA | NA | NA | NA | Heterozygous | c.883C>T, c.1118G>C | Q295X, R373P | 6, 8 | Itrazonazole | Alive | [30] |
| 6 | 2017 | NA | Algeria | Yes | 10 | 47 | | Skin, scalp, Nail, Lymph node and brain | Tinea corporis, Tinea capitis, Onychomycosis | TR | NA | NA | NA | NA | Homozygous | c.C865T | Q289X | 6 | Itraconazole | Alive | [23] |
| 7 | 2016 | VI-5 | Turkey | Yes | 8 | 55 | M | Skin, nail, lymph node | Tinea corporis, Onychomycosis | TV, TVV, TR | oral cavity | Candidiasis | CA, MFf | NA | | c.208C > T | R70W | 3 | griseofulvin,ketoconazole, fluconazole, itraconazole and terbinafine | NA | [12] |
| 8 | 2016 | NA | Afro American | NA | 8 | 12 | M | Skin | Tinea corporis | DR | abdominal mass | Aspergillosis | AF | | Homozygous | c.3G>C | M1I | NA | NA | NA | [50] |
| 9 | 2016 | V-2 | Turkish | NA | 8 | NA | M | Skin | Tinea corporis | TS | Meningitis, Tinea versicolor | Malasezia | NA | NA | Homozygous | NA | Q295X | 6 | NA | Alive | [55] |
| 10 | 2015 | NA | Egypt | No | 13 | NA | M | Skin, nail | Tinea corporis, Onychomycosis | TR | NA | NA | NA | NA | Homozygous | c.C865T | Q289X | 6 | posaconazole | Alive | [28] |
| 11 | 2015 | NA | Brazil | No | 11 | 24 | M | Skin | Tinea corporis | TM | Thrust | Candidiasis | CS | NA | Homozygous | c.302G>T | R101C | 3 | Itraconazole, Ketoconazole, posaconazole, terbinafine and amphotericin B | NA | [24] |
| 12 | 2013 | II-1 (Family 1) | Algeria | Yes | 6 | 75 | M | Skin, scalp, nails and lymph node | Tinea corporis, Tinea capitis, Onychomycosis | TV | NA | NA | NA | Non-insulin-dependent diabetes | Homozygous | c.C865T | Q289X | 6 | combination of griseofulvin and econazole | Alive | [9] |

https://doi.org/10.3390/jof12060451

| | | | | | | | | | | | | | | | | | | | | | |
|---|---|---|---|---|---|---|---|---|---|---|---|---|---|---|---|---|---|---|---|---|---|
| | | | | | | | | | | | | | | mellitus at 50 years | | | | | (Noneffective) then treated with Itraconazole (responded well) | | |
| 13 | 2013 | III-6 (Family 1) | Algeria | Yes | 2 | 29 | M | Skin, scalp, nails, lymph and brain | Tinea corporis, Tinea capitis, Onychomycosis | TV | NA | NA | NA | NA | NA | NA | NA | NA | itraconazole treatment not effective | Dead | [9] |
| 14 | 2013 | II-6 (Family 2) | Algeria | Yes | 9 | 40 | F | Skin, scalp, nails and lymph node | Tinea corporis, Tinea capitis, Onychomycosis | TR | NA | NA | NA | insulin-dependent diabetes mellitus at 35 years | Homozygous | c.C865T | Q289X | 6 | systemic corticosteroids, Griseofulvin, Itraconazole (during each relapse different treatment given at a time one agent given) | Alive | [9] |
| 15 | 2013 | II-1 (Family 3) | Algeria | Yes | 8 | 56 | M | Skin, scalp, and nails | Tinea corporis, Tinea capitis, Onychomycosis | TV | oral | Rucurrent oral Candidiasis | NA | NA | Homozygous | c.C865T | Q289X | 6 | Griseofulvin | Alive | [9] |
| 16 | 2013 | II-5 (Family 3) | Algeria | Yes | 8 | 34 | M | Skin, scalp, nails and lymph node | Tinea corporis, Tinea capitis, Onychomycosis | TV | NA | NA | NA | NA | NA | NA | NA | NA | Griseofulvin | Dead | [9] |
| 17 | 2013 | II-11 (Family 3) | Algeria | Yes | 8 | 41 | F | Nails | Onychomycosis | TV | recurrent thrush | Candidiasis | CA | NA | Homozygous | c.C865T | Q289X | 6 | Griseofulvin | Alive | [9] |
| 18 | 2013 | II-6 (Family 4) | Algeria | Yes | 19 | 43 | M | Skin, scalp, nails and lymph node | Tinea corporis, Tinea capitis, Onychomycosis | FH | NA | NA | NA | NA | Homozygous | c.C865T | Q289X | 6 | griseofulvin and fluconazole | Alive | [9] |
| 19 | 2013 | II-7 (Family 4) | Algeria | Yes | 21 | 40 | M | Skin, scalp, perineum and lymph node | Tinea corporis, Tinea capitis, Tinea cruris | FH | NA | NA | NA | NA | Homozygous | c.C865T | Q289X | 6 | initially griseofulvin then fluconazole then itraconazole and terbinafine | Alive | [9] |
| 20 | 2013 | II-4 (Family 4) | Algeria | Yes | NA | 28 | M | Skin, and scalp | Tinea corporis, Tinea capitis | FH | NA | NA | NA | NA | NA | NA | NA | NA | NA | Dead | [9] |
| 21 | 2013 | II-7 (Family 5) | Algeria | Yes | NA | 39 | M | Skin, scalp, and Lymph node | Tinea corporis, Tinea capitis | TV | NA | NA | NA | NA | Homozygous | c.C865T | Q289X | 6 | NA | Dead | [9] |
| 22 | 2013 | II-10 (Family 5) | Algeria | Yes | NA | 37 | F | Nails | Onychomycosis | DR | NA | NA | NA | NA | Homozygous | c.C865T | Q289X | 6 | NA | Alive | [9] |
| 23 | 2013 | II-6 (Family 6) | Morocco | Yes | NA | 40 | M | Skin, nail, bone and lymph node | Tinea corporis, Onychomycosis | TR | NA | NA | NA | NA | Homozygous | c.C301T | R101C | 3 | terbinafine,voriconazole, posaconazole, liposomal amphotericin B and combined treatment with terbinafine, | Alive | [9] |

| | | | | | | | | | | | | | | | | | | | | | |
|---|---|---|---|---|---|---|---|---|---|---|---|---|---|---|---|---|---|---|---|---|---|
| | | | | | | | | | | | | | | | | | | | voriconazole and interferon-γ | | |
| 24 | 2013 | II-3 (Family 6) | Morocco | Yes | Childhood | 49 | F | Scalp, nails | Tinea corporis, Onychomycosis | DR | NA | NA | NA | NA | Homozygous | c.C301T | R101C | 3 | NA | Alive | [9] |
| 25 | 2013 | II-6 (Family 7) | Tunisia | Yes | 6 | 91 | M | Skin, scalp, nails | Tinea corporis, Tinea capitis, Onychomycosis | DR | NA | NA | NA | NA | Homozygous | c.C865T | Q289X | 6 | NA | Dead | [9] |
| 26 | 2013 | III-1 (Family 7) | Tunisia | Yes | 12 | 44 | M | Scalp, nails | Tinea capitis, Onychomycosis | TR | NA | NA | NA | NA | Homozygous | c.C865T | Q289X | 6 | fluconazole followed by itraconazole | Alive | [9] |
| 27 | 2013 | III-4 (Family 7) | Tunisia | Yes | 5 | 52 | F | Skin, scalp, nails, lymph nodes | Tinea corporis, Tinea capitis, Onychomycosis | TR, TV | NA | NA | NA | NA | Homozygous | c.C865T | Q289X | 6 | Griseofulvine, ketoconazole and fluconazole | Alive | [9] |
| 28 | 2013 | II-1 (Family 8) | Tunisia | No | 6 | 62 | M | Skin, scalp, nails, lymph nodes | Tinea corporis, Tinea capitis, Onychomycosis | TR, TV | NA | NA | NA | NA | Homozygous | c.C865T | Q289X | 6 | griseofulvine, fluconazole, terbinafine and itraconazoleth en on 2nd episode by voriconazole | Alive | [9] |
| 29 | 2009 | II-2 (Family 2) | Iran | Yes | 45 | NA | F | Hand and Neck | Tinea corporis | NA | oral, vaginal | Candidiasis | NA | intermittent aphthous lesions, type 2 diabetes mellitus, and nephrolithiasis | Heterozygous | C→T | Q295X | 6 | NA | NA | [10] |
| 30 | 2009 | II-2 (Family 1) | Iran | Yes | Childhood | NA | F | Chest and Neck | Tinea corporis | NA | oral, vaginal | Candidiasis | NA | NA | Homozygous | C→T | Q295X | 6 | NA | NA | [10] |
| 31 | 2009 | II-3 (Family 5) | Iran | Yes | Childhood | NA | M | NA | Tinea corporis | NA | NA | NA | NA | NA | Homozygous | C→T | Q295X | 6 | Local treatment | NA | [10] |

**TR- *Trichophyton rubrum*, TT- *Trichophyton tonsurans*, TV-*Trichophyton violaceum*, TVV- *Trichophyton verrucosum*, TM- *Trichophyton mentagrophytes*, TS- *Trichophyton* spp., MF- *Microsporum ferrugineum*, DR- Dermatophytes, FH- Fungal hyphae, CA- *Candida albicans*, CS- *Candida* spp., MI- *Mucor irregularis*, AF- *Aspergillus fumigatus*, AFl- *Aspergillus flavus*, MFf- *Malassezia furfur*, M- Male, F- Female, NA- No information, c- Coding DNA sequence, p-Protein.**

***Pedigree notation indicates the position of the affected individual within a family: Roman numerals denote the generation (I, II, III), and Arabic numerals indicate the individual within that generation. 'Family' numbers (e.g., Family 1, Family**
